# Validation of computed tomography for measuring lung weight

**DOI:** 10.1186/s40635-014-0031-0

**Published:** 2014-12-05

**Authors:** Alessandro Protti, Giacomo E Iapichino, Marta Milesi, Valentina Melis, Paola Pugni, Beatrice Comini, Massimo Cressoni, Luciano Gattinoni

**Affiliations:** Dipartimento di Anestesia, Rianimazione ed Emergenza Urgenza, Fondazione IRCCS Ca′ Granda - Ospedale Maggiore Policlinico, Via F. Sforza 35, 20122 Milan, Italy; Dipartimento di Fisiopatologia Medico-Chirurgica e dei Trapianti, Università degli Studi di Milano, Via della Commenda 16, 20122 Milan, Italy

**Keywords:** Computed tomography, Lung weight, Ventilator-induced lung injury, Pulmonary oedema

## Abstract

**Background:**

Lung weight characterises severity of pulmonary oedema and predicts response to mechanical ventilation. The aim of this study was to evaluate the accuracy of quantitative analysis of thorax computed tomography (CT) for measuring lung weight in pigs with or without pulmonary oedema.

**Methods:**

Thirty-six pigs were mechanically ventilated with different tidal volumes and positive end-expiratory pressures that did or did not induce pulmonary oedema. After 54 h, they underwent thorax CT (CT_*in vivo*_) and were then sacrificed and exsanguinated. Fourteen pigs underwent a second thorax CT (CT_post-exsang._) after exsanguination. Lungs were excised and weighed with a balance (balance_post-exsang._). Agreement between lung weights measured with the balance (considered as reference) and those estimated by quantitative analysis of CT was assessed with Bland-Altman plots.

**Results:**

One animal unexpectedly died before CT_*in vivo*_. In 35 pigs, lung weight measured with balance_post-exsang._ was 371 ± 184 g and that estimated with CT_*in vivo*_ was 481 ± 189 g (*p* < 0.001). Bias between methods was −111 g (−35%) and limits of agreement were −176 (−63%) and −46 g (−8%). Measurement error was similar in animals with (−112 ± 45 g; *n* = 11) or without (−110 ± 27 g; *n* = 24) pulmonary oedema (*p* = 0.88). In 14 pigs with thorax CT after exsanguination, lung weight measured with balance_post-exsang._ was 342 ± 165 g and that estimated with CT_post-exsang._ was 352 ± 160 g (*p* = 0.02). Bias between methods was −9 g (−4%) and limits of agreement were −36 (−11%) and 17 g (3%). Measurement errors were similar in pigs with (−1 ± 26 g; *n* = 11) or without (−12 ± 7 g; *n* = 3) pulmonary oedema (*p* = 0.12).

**Conclusions:**

Compared to the balance, CT obtained *in vivo* constantly overestimated the lung weight, as it included pulmonary blood (whereas the balance did not). By contrast, CT obtained after exsanguination provided accurate and reproducible results.

## Background

During ventilator-induced lung injury and acute respiratory distress syndrome, lung weight increases as a result of inflammatory pulmonary oedema [1,2]. Exact quantification of this phenomenon can help diagnosis, prognosis and even therapy. For instance, patients with heavy (and largely recruitable) lungs benefit the most from use of high positive end-expiratory pressure [3] or prone position [4].

One method for estimating lung weight *in vivo* is quantitative analysis of thorax computed tomography (CT). Lungs are selected on CT images and their total (gas and tissue) volume and physical density (that presumably reflects relative content of gas and tissue) are measured. Lung tissue volume is derived from these two parameters. Lung tissue weight is computed assuming that the density is 1 g/ml [5].

Selection of region of interest is crucial for quantitative analysis of CT. It is usually performed manually, based on individual judgement, and is prone to errors especially when contours are not linear and content is not homogeneous [6]. Inaccuracy is indeed less than 1% for phantoms with regular shape [7] but as high as 10% for objects with complex geometry, including human organs [8]. Oedema may act as an additional confounder as it deforms anatomy and increases inhomogeneity.

The aim of this study was to validate quantitative analysis of CT (against gravimetry) for measuring lung weight in pigs with or without pulmonary oedema.

## Methods

This is a sub-study of our past [9,10] and present works. Experiments complied with international recommendation [11] and were approved by the Italian Ministry of Health.

### Validation of CT performed in living animals (CT_*in vivo*_) for measuring lung weight

Thirty-six consecutive healthy female pigs (23 ± 3 kg) were mechanically ventilated with different tidal volumes and positive end-expiratory pressures under general anaesthesia (propofol [5 to 10 mg/kg/h IV] and medetomidine [2.5 to 10.0 μg/kg/h IV]) and paralysis (pancuronium bromide [0.3 to 0.5 mg/kg/h IV]). After 54 h, thorax CT (CT_*in vivo*_) was obtained. Animals were then sacrificed (KCl 40 mEq IV) and exsanguinated through an incision in the inferior vena cava. Lungs were excised *en bloc*, dissected from the trachea, main bronchi and hilar lymph nodes, and weighted with a balance (balance_post-exsang._) within 30 to 60 min from CT_*in vivo*_. The balance (Bizerba Maxima Super Elox, Bizerba, Milan, Italy) underwent periodical tests with calibrated weights. Percentage error (see below) was always <1%. Ventilator-induced pulmonary oedema was diagnosed if balance_post-exsang._ lung weight exceeded 400 g, the upper limit for healthy pigs of similar sex and weight [1,9,10].

One animal unexpectedly died before we could obtain thorax CT_*in vivo*_. It was immediately exsanguinated and included in the second part of the study (see below).

### Validation of CT performed after exsanguination (CT_post-exsang._) for measuring lung weight

Fourteen (out of 36) animals underwent a second thorax CT (CT_post-exsang._) once exsanguinated. The volume of blood removed was always quantified, whereas density (g/ml) was measured in three cases. Lungs were then excised and weighted with the balance (and pulmonary oedema was eventually diagnosed) as above.

### Quantitative analysis of CT

CTs were obtained at 0 cmH_2_O of airway pressure with the following settings: collimation, 5 mm; interval, 5 mm; bed speed, 15 mm/s; voltage, 140 kV; and current 240 mA (Lightspeed QXi, GE Healthcare, Madison, WI, USA). Quality controls were performed every month using standard phantoms.

Experienced operators manually countered the lung profile excluding proximal airways, large vessels and lymph nodes, mediastinum, muscles and bones and pleural effusions (Maluna 3.15, University Hospital of Goettingen, Germany).

For each voxel of interest, tissue weight was

$$ \mathrm{Voxel}\ \mathrm{tissue}\ \mathrm{weight}=\left[1\hbox{--} \left(\mathrm{Voxel}\ \mathrm{density}/-1,000\right)\right]\times \mathrm{Voxel}\ \mathrm{volume} $$

Voxel density was expressed in Hounsfield units (HU), with values of −1,000, 0 and +1,000 HU assigned to air, lung tissue (including parenchyma, blood and water) and bone, respectively. Voxel volume was 1.8 mm^3^.

Lung tissue weight was the sum of the weight of all selected voxels [5].

### Repeatability and reproducibility of quantitative analysis of thorax CT

Thorax CTs_*in vivo*_ of one animal with and one animal without pulmonary oedema were analysed thrice by the same operator (repeatability) and once by three different operators (reproducibility).

### Statistical analysis

Normality of data distribution was verified with the Shapiro-Wilk test. Results are reported as means ± standard deviations. Difference between groups was assessed with Student's *t* test or Mann-Whitney rank sum test. Agreement between reference (balance; gravimetry) and test (quantitative analysis of CT) methods was studied with Bland-Altman plots and analysis of correlation [12]. Percentage error was the ratio between the limits of agreement and reference measurement. Repeatability and reproducibility were expressed as coefficients of variation, the ratio between standard deviations and means. Statistical significance was defined as *p* < 0.05 (SigmaPlot 11.0, Jandel Scientific Software, San Jose, CA, USA).

## Results

### Validation of CT performed in living animals (CT_*in vivo*_) for measuring lung weight

**Figure 1:**
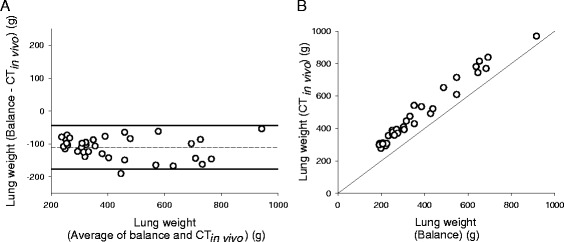
**Agreement and association between balance (balance**_**post-exsang**._ **) and CT performed in living animals (CT**_***in vivo***_ **) for measuring lung weight. (A)** Plot of individual differences between lung weights measured with balance_post-exsang._ (reference) and CT_*in vivo*_ against their means. Dashed line refers to overall mean difference (bias), and solid lines correspond to 95% limits of agreement (mean difference ± 1.96 standard deviation). **(B)** Individual lung weights measured with balance_post-exsang._ and CT_*in vivo*_, with line of identity [CT_*in vivo*_ lung weight = 106 + 1.01 × (balance_post-exsang._ lung weight)].

In 35 pigs (11 with pulmonary oedema), lung weight was 371 ± 184 g when measured with balance_post-exsang._ and 481 ± 189 g (*p* < 0.001) when estimated with quantitative analysis of CT_*in vivo*_. Bias was −111 g (−35%) and limits of agreement were −176 (−63%) and −46 g (−8%) (Figure 1a). Percentage error was 35% and coefficient of correlation was 0.97 (*p* < 0.0001) (Figure 1b).

On average, balance_post-exsang._ lung weight was 606 ± 140 g in animals with pulmonary oedema (*n* = 11) and 263 ± 55 g in those without pulmonary oedema (*n* = 24). Absolute measurement errors were not associated with (mean) lung weight (*r* = 0.16) (*p* = 0.36) and did not differ between animals with (−112 ± 45 g) or without (−110 ± 27 g) pulmonary oedema (*p* = 0.88). Diversely, relative measurement errors were smaller in animals with (−19%; limits of agreement −36% and −3%) than in those without (−43%; limits of agreement −61% and −24%) pulmonary oedema (*p* < 0.01).

### Validation of CT performed after exsanguination (CT_post-exsang._) for measuring lung weight

**Figure 2:**
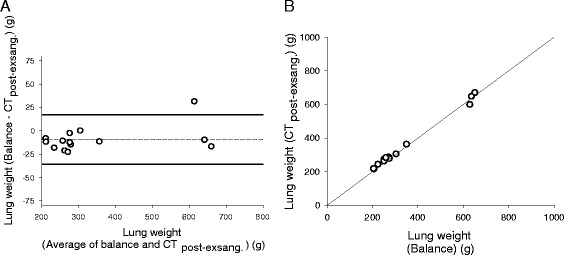
**Agreement and association between balance (balance**_**post-exsang.**_ **) and CT performed after exsanguination (CT**_**post-exsang.**_ **) for measuring lung weight. (A)** Plot of individual differences between lung weights measured with balance_post-exsang._ (reference) and CT_post-exsang._ against their means. Dashed line refers to overall mean difference (bias), and solid lines correspond to 95% limits of agreement (mean difference ± 1.96 standard deviation). **(B)** Individual lung weights measured with balance_post-exsang._ and CT_post-exsang._, with line of identity [CT_post-exsang._ lung weight = 22 + 0.96 × (balance_post-exsang._ lung weight)].

In 14 pigs (three with pulmonary oedema), lung weight was 342 ± 165 g when measured with balance_post-exsang._ and 352 ± 160 g when estimated with quantitative analysis of CT_post-exsang._ (*p* = 0.02). Bias was −9 g (−4%) and limits of agreement were −36 (−11%) and 17 g (3%) (Figure 2a). Percentage error was 15% and coefficient of correlation was 0.99 (*p* < 0.0001) (Figure 2b).

On average, balance_post-exsang._ lung weight was 640 ± 11 g in animals with pulmonary oedema (*n* = 3) and 261 ± 42 g in those without pulmonary oedema (*n* = 11). Absolute measurement errors were not associated with (mean) lung weight (*r* = 0.41) (*p* = 0.15) and did not differ between animals with (−1 ± 26 g) or without (−12 ± 7 g) pulmonary oedema (*p* = 0.12).

The volume of blood aspirated during exsanguination was 1,049 ± 283 ml. The density of blood (measured in three animals) was 1.01 g/ml.

In 13 animals that underwent both CTs, CT_*in vivo*_ overestimated lung weight by 105 ± 32 g compared to CT_post-exsang._ and by 117 ± 32 g compared to the balance (*p* = 0.23).

### Repeatability and reproducibility of quantitative analysis of thorax CT

Coefficients of intra- and inter-subject variability were 2.4% and 4.2%, respectively, for healthy lungs and 2.2% and 0.3%, respectively, for oedematous lungs.

## Discussion

Compared to gravimetry, quantitative analysis of thorax CT accurately measures the weight of lungs of exsanguinated animals (CT_post-exsang._: bias −4%; percentage error 15%). However, it largely overestimates the weight of lungs of living animals (CT_*in vivo*_: bias −35%; percentage error 35%) as it includes pulmonary blood.

Other authors have shown that CT properly quantifies the weight of inanimate objects [8], artificial lung models [13], exsanguinated [14] and frozen [15] lungs and surgically excised pulmonary lobes [16]. Results acquired *in vivo* can be less accurate. For instance, in previous studies, the weight of the right hepatic lobes differed by 20% to 35% when measured pre-operatively with CT (*in vivo*) or intra-operatively with a balance (*ex vivo*). One plausible explanation for this discrepancy is blood volume: measurements taken before surgery included blood, whereas those obtained after graft procurement did not [17,18]. In our study, lung weight measured with CT_*in vivo*_ (before exsanguination) exceeded by around 100 g that obtained with CT_post-exsang._ or the balance (after exsanguination). Pulmonary blood is around 5 ml/kg of body weight in pigs [19] and represents 10% to 15% of the total blood volume in mammals [20] (normal values in humans: 220 to 270 ml/m^2^ of body surface area [21]). Therefore, it likely approximated 100 ml in 20-kg pigs that exsanguinated, on average, 1 l of blood. Since blood density was 1 g/ml, pulmonary blood weight of animals included in our experiments was probably around 100 g and thus explains the bias reported above. Of note, measurement error was the same even in animals with (inflammatory) pulmonary oedema as if changes in lung weight induced by mechanical ventilation mainly reflected changes in extravascular water content [22]. As a consequence, relative measurement error was as low as 19% in animals with pulmonary oedema.

In line with the model described so far, differences between computed tomography and gravimetry virtually disappeared when they both measured blood-free lung weights (CT_post-exsang._ vs. balance_post-exsang._). To further corroborate this finding, two other animals underwent thorax CT_*in vivo*_ and were then sacrificed but not exsanguinated to allow intravascular blood clotting. Two hours later, lungs were excised with minimal blood loss. As expected, results of quantitative analysis of thorax CT_*in vivo*_ differed by only 24 g (5%) and 11 g (3%) from those obtained with the balance as both methods measured the weight of blood-filled lungs.

Some limitations of the study deserve a comment. First, sample size was limited due to inconstant availability of CT, some unexpected early deaths and completion of research projects to which animals were originally assigned. Accuracy of CT_post-exsang._ was evaluated only in three animals with pulmonary oedema, and therefore, results should be interpreted with caution. Second, the volume of blood withdrawn from animals was quite variable, possibly because heparin was not used to prevent post-mortem thrombosis. This may be the reason why percentage error of lung weight estimated with CT_*in vivo*_ was as high as 35%. Third, the use of thorax CT in clinical practice requires transfer of patients to radiology [23] and exposure to ionising radiations [24]. In addition, quantitative analysis is complex and time consuming. However, low-dose exams [25] and simplified analysis [26,27] may limit risks and expense. Moreover, quantitative analysis of thorax CT does not only measure lung weight but also characterises patients with acute respiratory failure [28], assesses distribution of ventilation [29], quantifies pulmonary inhomogeneities [30] and predicts response to therapy [31].

## Conclusions

When performed on living subjects, quantitative analysis of thorax CT scan constantly overestimated lung weight as it included pulmonary blood (whereas the balance did not). In bloodless conditions, it provided accurate, reproducible and repeatable results.

## Abbreviations

CT: computed tomography
Post-exsang.: following exsanguination
HU: Hounsfield unit
IV: Intravenous
KCl: Potassium chloride
